# PARTNER: A qualitative study on academic and community hospitals partnerships to optimize outbound patient transfers and capacity

**DOI:** 10.1002/jhm.70141

**Published:** 2025-08-04

**Authors:** Ruby Marr, Sachita Shrestha, David Haughey, David Bozaan, Dahlia Rizk, John P. Downs, Rafina Khateeb

**Affiliations:** ^1^ Department of Internal Medicine, Division of Hospital Medicine University of Michigan Health Ann Arbor Michigan USA; ^2^ Dartmouth Hitchcock Medical Center Lebanon New Hampshire USA; ^3^ Mount Sinai Health System New York New York USA; ^4^ UNC Health Chapel Hill North Carolina USA

## Abstract

**Background:**

While interhospital patient transfers are common, most existing literature centers on transfers to academic medical centers (AMCs) and tertiary/quaternary care hospitals for a higher level of care. With the growing trend of healthcare system consolidation and formation of regional affiliations, many systems now coordinate admissions and capacity on a broader system‐wide scale. As a result, some AMCs have developed programs to facilitate outbound transfer to regional affiliate hospitals. This shift prompted our study.

**Objectives:**

Our study aims to understand the current landscape of outbound transfer programs and to identify key facilitators and barriers to successful implementation, resultant operational successes, and future aspirations.

**Methods:**

We conducted semi‐structured qualitative interviews with AMCs across the United States that have an affiliation with another institution. Affiliation is defined as “an agreement to collaborate on an initiative or to provide a specific service together. This may involve local, regional or national partners.” Data were analyzed using inductive and deductive methods in Nvivo software.

**Results:**

We conducted interviews with 12 AMCs from all four major United States regions ranging from 550 to 1700 beds. We identified five key themes from the study and included: (1) transfer activity, (2) barriers to implementation, (3) facilitators to implementation, (4) successes, and (5) aspirations.

**Conclusion:**

This study highlights the vital role of patient transfer programs in ensuring high‐quality care and managing capacity within AMCs and healthcare systems. Successful implementation depends on addressing inefficiencies, fostering collaboration, and promoting equitable care. Prioritizing these factors can improve patient outcomes and alleviate capacity constraints, enabling timely access to necessary care.

## INTRODUCTION

Patient transfers between hospitals are routine in healthcare, with an estimated 3.3 million ED to hospital transfers annually.^1^ A significant portion of these involves transfers to academic medical centers (AMCs) or tertiary/quaternary care centers for specialized services.^2^ Market forces contribute to the growing volume of inbound transfers to AMCs. Hospital consolidation has accelerated, with over 2000 mergers in the past three decades.^3^ Often driven by financial pressures, health systems have centralized services into “centers of excellence” to reduce redundancy and better allocate resources.^4^ Conversely, high‐volume hospitals—particularly during surge periods such as the coronavirus disease 2019 (COVID‐19) pandemic—have relied on less‐burdened community partners to manage bed capacity. This strategy allows AMCs to preserve high‐acuity beds for complex, higher revenue cases, while financially supporting community affiliates by increasing their census.^5^ Despite the growth of outbound transfers from AMCs to community partners, there is limited research exploring the operational factors that influence their success.

The PARTNER study has five aims: (1) explore transfer practices from AMCs to regional affiliates*, (2) understand the purpose of these transfers, (3) identify barriers and facilitators, (4) examine successes and aspirations, and (5) share operations to guide other AMCs considering similar transfers. *Affiliation is defined as a collaboration agreement for specific services, involving local, regional, or national partners. The goal is to provide a practical roadmap for health systems seeking to enhance outbound transfer strategies to community partners.

## METHODS

This qualitative, multicenter study used convenience sampling to gather perspectives from AMC representatives involved in patient transfer. Participants were recruited via two well‐established national groups: the Society of Hospital Medicine's Interhospital Transfers Special Interest Group and HOMERuN, through an email invitation linked to an eligibility survey on Qualtrics. The survey asked: (1) Does the AMC have affiliations with one or more community hospitals (clinical, educational, or research)? (2) Does the AMC transfer or refer acute medicine patients to affiliated community hospitals? Institutions answering “Yes” to both were included. Of 23 responding AMCs, 13 met eligibility, and 12 institutions with 17 representatives participated in semi‐structured Zoom interviews.

Authors (R. M., R. K., D. B., and D. H.) conducted interviews with the participants via Zoom video conferencing. Before the interview, participants were provided with the semi‐structured interview guide (Appendix S1) and supplied written consent to participate. The interviews lasted from 35 to 60 min and occurred August 2023 through January 2024. They were recorded and transcribed. The authors (R. M., R. K., D. H., and D. B.) reviewed transcripts for accuracy. Transcripts were imported to Nvivo software for analysis. Using thematic analysis, the authors (S. S., R. M., R. K., and D. B.) separately coded the first four transcripts inductively. Each transcript was coded by two study team members who then met to discuss and compare coding. They reviewed each code to identify agreements and disagreements. The level of agreement between the coders was assessed in Nvivo using Cohen's kappa coefficient. For codes that initially did not match, discussions were held to reach a consensus, involving a re‐evaluation of relevant transcript sections and a review of the coding framework for clarity. After reaching consensus, the team recoded the transcripts to incorporate agreed upon changes. The process was repeated iteratively until each transcript achieved at least 80% inter‐rater reliability. The team then finalized the codebook that was used to code the remainder of the transcripts and categorized the codes into categories and subcategories to generate themes. The Institutional Review Board of the University of Michigan Medical School (IRBMED) approved this study as an exempt study (HUM00225766).

## RESULTS

Of the 23 AMCs that responded to our eligibility survey, 13 met the eligibility criteria. One institution was unable to be scheduled for an interview successfully. We conducted semi‐structured interviews with 17 participants from 12 institutions. Participants included Hospital Medicine administrators, Hospital Medicine leaders with varying titles, transfer center leaders with varying titles, community affiliate relations leaders, and an Emergency Room director.

Five distinct domains emerged: (1) Transfer activity, (2) Barriers, (3) Facilitators, (4) Successes, and (5) Aspirations (Table 1).

**Table 1:** Themes, category, and subthemes.

Theme	Category	Subthemes
Transfer activity	Definition and organizational structure	Transfer destination
		Number of affiliates
		Transfer type
		Affiliate relationship
	Purpose	Increase capacity
		Access to specialized care
	Patient selection	Diagnoses
		Populations
		Inclusion and exclusion criteria
		Identification
	Metrics	
Barriers	Conflicting priorities/interests	Competing demands
		Capacity across institutions
		Interplay between level of care and desired patient populations
	Implementation and operational challenges/gaps	Triage complexity
		Implementation/buy‐in
		Weak management systems
	Resources impacting operations	Staffing
		Transportation
Facilitators	Institutional factor	Common electronic medical record
		Centralized transfer center
		Affiliate is closer to AMC
		Marketing materials
	Provider factor	Shared provider staffing
		Empowerment of dedicated role
	Patient factor	Preference to be closer to home or in a smaller hospital
Successes	Improved care delivery and improved patient/provider satisfaction	Patient and or family satisfaction
		Provider satisfaction
		Quality improvement
		Special population care
	Growth	Resource growth at affiliate
		Growth in services offered at affiliate
	Improved relationships	Institutional relationship
		Educational or faculty development benefit
		Research benefit
	Improved capacity management	Capacity management
Aspirations	Growth of affiliations	
	Expansion of populations/conditions	
	Improvement of operations/management system	
	Growth of services to match patient's preferred geographic location	

Abbreviation: AMC, academic medical center.

### Transfer activity

#### Definition and organizational structure

**Figure 1:**
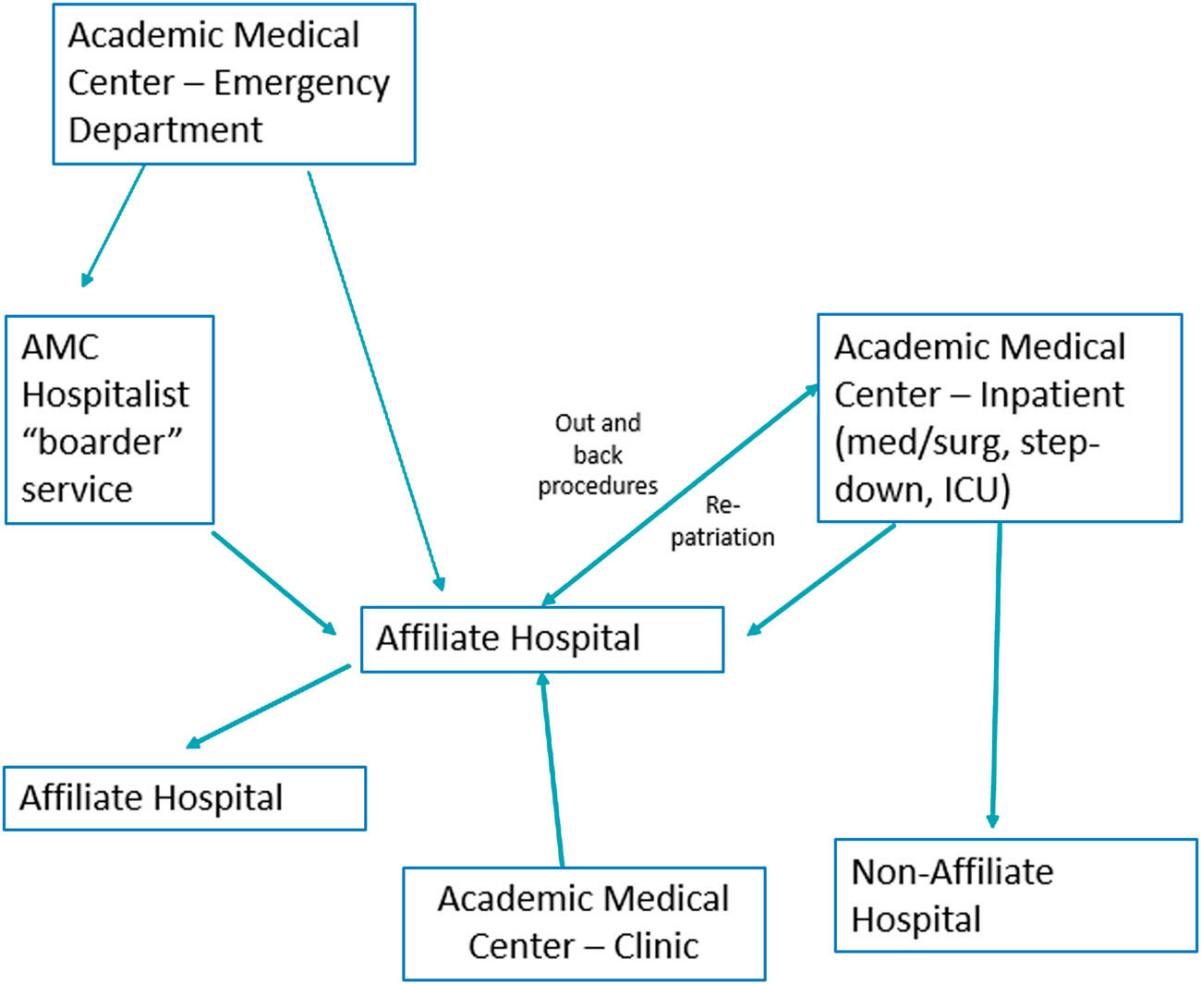
Patient transfer overview. AMC, academic medical center.

Outbound transfers from AMCs varied in form (see Figure 1). All programs transfer patients from the primary AMC to an affiliated hospital within the same or a different health system. The number of affiliates per AMC ranged from 2 to 16, though most transfers were concentrated in one to three nearby affiliates.

Nine programs transferred patients from the AMC Emergency Department (ED). Eight programs transferred inpatients, and three programs transferred intensive care unit (ICU) and/or step‐down patients to an affiliate. Nine programs included affiliates that were AMC‐owned, and transfers were perceived as easier when affiliates share a tax ID and legally act as the same entity. One program transferred patients to nonaffiliate hospitals.

#### Purpose of the transfer programs

Most transfer programs aimed to increase ED and inpatient capacity at the AMC by utilizing available beds across affiliates, a process known as “level loading.” This ensured “the right bed, for the right patient, at the right time” (P9). Another goal was improving access to specialized care at affiliates, such as surgery centers of excellence.

#### Patient selection

Two programs transferred pediatric patients. Some focused on specific diagnoses (e.g., heart failure exacerbation), while others frequently transferred certain populations (e.g., sickle cell crisis, chronic pain, alcohol withdrawal), raising equity concerns. Policies on “placement patients” (awaiting postacute care) varied—some programs targeted them, others excluded them.

Many programs used affiliate exclusion criteria (unavailable services at the affiliate). Some prioritized transfers near the patient's home zip code. One program employed an artificial intelligence (AI) model to screen ED patients for appropriateness to transfer to affiliates, while another relied on ED case managers monitoring an electronic medical record (EMR) list.

#### Metrics

All programs tracked transfer volume (see Table 2), with most also monitoring back‐transfers to the AMC. One program reviewed each back‐transfer in weekly quality improvement (QI) meetings, while another tied patient transfer count to individual hospitalists as a clinical productivity measure.

**Table 2:** Patient transfer activity.

Participant # & AMC Region	Size^a^	Transfer type(s)^b^	Adults	Pediatrics	# affiliates	# patients per month	Unified transfer center	Dedicated triage staff	Shared EMR	Ask patient consent for transfer
1‐Northeast	1000	abcd	Yes	No	16	132	Yes	Yes	Yes	No
2‐South	1000	ab	Yes	No	10	4	Yes	Yes	Yes	Yes
3‐Northeast	1500	ae	Yes	Yes	2	150	Yes	Yes	Yes	Yes
4‐Northeast	1100	ag	No	Yes	3	5	n/a	Yes	No	Yes
5‐West	550	abdf	Yes	No	13	200	Yes	Yes	Yes	Yes
6‐West	675	a	Yes	No	2	100	n/a	Yes	Yes	No
7‐Midwest	1700	b	Yes	No	7	83	Yes	Yes	Yes	Yes
8‐Midwest	1400	bc	Yes	No	2	30–100	Yes	Yes	Yes	Yes
9‐Northeast	1000	bcg	Yes	No	1	2	Yes	No	Yes	Yes
10‐Northeast	650	abg	Yes	No	9	100	Yes	Yes	Yes	Yes
11‐Midwest	1000	ad	Yes	No	2	1	Yes	Yes	Yes	Yes
12‐Midwest	1125	abg	Yes	No	3	120	Yes	Yes	No	Yes

Abbreviations: AMC, academic medical center; ED, Emergency Department; EMR, electronic medical record.

^a^ Rounded to nearest 25 inpatient beds.

^b^ a (AMC ED to affiliate), b (AMC inpatient to affiliate), c (out and back procedures), d (repatriation AMC to affiliate), e (AMC clinic to affiliate), f (AMC to non‐affiliate), g (affiliate to affiliate transfers orchestrated by AMC transfer center).

Tracked metrics varied including admission diagnoses, inpatient/observation status, posttransfer length of stay, patient/provider satisfaction, escalation of care needs, aborted transfers, affiliate acceptance rates, and transfer impact on ED boarding. One program shared data with affiliates, comparing AMC‐to‐affiliate transfers with affiliate‐to‐AMC requests. Participants stressed the importance of monitoring for patient harm.

I think the most important metrics we can use is are we harming our patients? Where's the best care delivered?… Where's the best place this patient can receive their care…make the decision based on that. P10

### Barriers to implementation

Study participants described many barriers, including conflicting priorities or interests between institutions, implementation and operational gaps or challenges, as well as how the lack of resources affected operations.

#### Conflicting priorities/interests

Participants noted that the needs and priorities of AMCs and their affiliates can directly conflict.

We actually even have rules. We're not allowed to send people over to these [Affiliate] hospitals that are placement only at that point, which makes it really difficult and, in my opinion, not an effective use of our hospital system. P5

Conflicting priorities emerged around two patient groups: long length‐of‐stay (LLOS) patients and those with chronic conditions. LLOS patients are considered lower complexity and suitable for community hospitals, affiliates often avoid accepting them. Patients with chronic pain or substance use disorders (SUDs) are frequently transferred, sparking concerns about selection bias. Provider dissatisfaction in managing these populations was mentioned.

One of the side effects we're seeing from this, if you look at the patients who are getting transferred, you run into real equity issues. Patients with sickle cell anemia with pain crisis. Otherwise, with chronic pain, chronic pancreatitis, drug use … We're sending most of our alcohol withdrawal. I worry about that. I don't know what to do about it, but I'm worried about it. P7

#### Implementation and operational challenges/gaps

Participants highlighted operational challenges in coordinating capacity between systems. Specifically, there were difficulties in understanding inclusion and exclusion criteria for transfer to affiliates given the many nuances.

I'd say the most common problem we get is the [Affiliate] is small enough where while we do have a lot of resources 24/7, 365 days a year, the breadth of procedures that they perform for said resource varies sometimes from week to week or day to day, depending on which IR physician is on, which pulmonologist is on, et cetera, which orthopedist. P5

A key implementation challenge was securing buy‐in from patients and families. They perceived AMCs as providing superior care and outcomes, making them reluctant to accept transfers to affiliate institutions.

I think the biggest factor is their affinity to the [AMC] brand, it plays a big role in the percentage of how many agree to go. We hear a lot from patients that ‘I came here to experience the [AMC] difference. I really don't want to go anywhere else, I could have driven to those other hospitals’. Sometimes we're able to overcome that by explaining that it's the same doctors or hospitalists who would see you here. They're the same ones that would see you there. But sometimes we're not. P4

Participants also highlighted challenges in gaining provider buy‐in at both AMCs and affiliates for patient transfers. Resistance to change and adherence to traditional practices were cited as key barriers to implementation.

One of the limitations is comfort with management, for example, of acute kidney injury and, let's say, pyelonephritis, in a patient with a kidney transplant. It's really not much different. Once you figure out they're not obstructed, and they're not rejecting right? It's really not much different than what we do in regular pyelonephritis. But until you've done it dozens of times you don't know that. And so our community hospitals don't know that stuff. And so they're very uncomfortable taking care of these patients. You gotta have somebody to say yes, there, and you have to have the nursing unit say, yes, the administrative Nursing Supervisor say yes, and the physician say yes. P7

Participants highlighted weak and underdeveloped management systems as a major operational challenge. Institutional EMRs and transfer centers lack the sophistication to identify eligible patients for transfer, requiring manual case reviews. Additionally, systems fail to prioritize beds at affiliates, leading to parallel admission processes at both the AMC and affiliate. Real‐time capacity updates across institutions are lacking, making transfer availability constantly shifting and unpredictable.

We have three community hospitals, home hospital and observation status. All these pathways are happening simultaneously, not in a stepwise fashion. The search for Community Hospital is triggered by the bed request. So at the same time, our admitting department is looking for an inpatient bed. It's a race to see who wins, and it's a slow race on both fronts. We want the community hospitals to win… And I think we haven't perfected the system to solve those challenges. P9

Subjectivity in determining transfer eligibility complicates the transfer process. The lack of clear criteria for which clinical states or diagnoses qualify for transfer creates operational challenges.

I've noticed some [AMC] specialists would like a case to come to [Affiliate], or they agree. Yet what happens is, the patient will come over, and the consultants are like, why did we take this patient? P4

#### Resources impacting operations

Lack of staffing as well as transportation impacted operations.

We did have at some point, some challenge with staffing during peak admission time at [Affiliate]. We've been able to resolve that with adding staff. I would also say one other major opportunity or challenge that we had is in the smaller hospitals not all of the nursing competencies and resources were there. And we, you know, really worked hard to fix it. Bring in new nursing. P12

Patients’ delays in care were impactful.

It has been a challenge, we've had so many delays that have been an impediment to getting this program to work. You can imagine, part of the call is like. ‘Oh, well, we could get you to [Affiliate], and you'll have a private room there in like 2 h, or you could wait in our ED for 2 days, and who knows if you'll ever get it?’ ‘Oh, yeah, sign me up.’ And then wait for 12 h, because the ambulance doesn't come, and they're like well why did I agree to do that? I might as well have clocked that time towards getting a bed here, you know. So that's kind of been a hard thing. P9

### Facilitators

We grouped facilitating factors into three different levels: institutional, provider, and patient level factors.

#### Institutional factors

The majority of participants emphasized the value of a shared EHR system in managing admissions and referrals. It provided real‐time access to patient data (e.g., medication administration, therapy dates) and enabled efficient communication via Epic secure chat for transfer sign‐out.

Because we share the same EHR, they're able to see when the last dose of antibiotics were, or they're able to see the last physical therapy note. I think that has helped streamline things…. Now that we have secure chat, that has lowered that barrier a lot. They can just secure chat the provider who wrote the note that day or the day before. P6

A centralized transfer center was a key facilitator, identifying affiliate site capabilities and documenting AMC‐affiliate transfer communications. Participants noted it eased the burden on front‐line providers, allowing them to focus on patient care.

The nice thing is, it all functions through our transfer center. So any patient would go through the transfer center, and we have a hospitalist that staffs that along with the nurse and they would together decide if the patient sounds appropriate for the [Affiliate], and then they facilitate that once that decision has been made. P11

Participants noted that the distance between the AMC and the affiliate site played a significant role in transfer decisions. If the affiliate site is closer to the AMC, patients are more likely to be transferred there.

So our closest affiliate is our community hospital which is about 15 miles down the road. We do our highest volume [transfer]. P2

Two institutions used marketing tools such as posters and brochures to inform patients about affiliate services and connection postdischarge back to the AMC clinic. This helped align expectations and facilitated patient consent for transfers.

The third tool that we use is marketing tools to help with patient consent. So we have posters in every ER room. They sort of prepare the patient that you could be admitted to many different sites… We have brochures that get handed out to the patient by whomever is speaking to them about going to the affiliate site. It has information about who will be caring for them, how we'll be connecting them back to their specialists and providers at the AMC. What to expect in terms of transportation, and so forth. Giving them as much information upfront as possible, so we can be on the same page in terms of expectations. P12

#### Provider factors

Shared providers between AMCs and affiliates streamlined transfers. Their familiarity with both facilities’ protocols and resources enhanced communication, coordination, and trust, ensuring continuity of care and a smoother transfer process.

We have a better relationship with [Affiliate]. We can accept without having an accepting physician on the affiliate side. Some of our [Affiliate] predominance hospitalists also work as the intake physician at [AMC]. We're pretty well integrated, and we know their capacity, their specialties really well. P5

A dedicated hospitalist or team overseeing transfers improved efficiency. Empowering this team helped overcome transfer barriers and ensured appropriate patient placement.

We have implemented a central hospitalist. I have done that over the last year and a half in a pilot that's been extremely effective. It used to take us 4 or 5 hours to connect one hospitalist to another hospitalist at any given site because the front line providers are extremely overwhelmed and very busy, especially in the off hours. By implementing a dedicated person that's just responsible for reviewing the chart, assessing the suitability, making sure these cases are stable for transfer, and then actually getting and giving the turnover to the receiving. We've cut that time down dramatically […] from hours to 6 min. P4

#### Patient factors

Participants highlighted patient‐related factors that facilitated the transfer process. Patients were more likely to accept transfer if the affiliate site was closer to their home, as this proximity allowed them to receive psychosocial support from family and friends. Some participants mentioned that patients preferred the affiliate site because it is quieter and logistically easier or hassle‐free compared to the AMC location.

We usually got it [consent] for the patient and family that came from two hours away, five hours away, eight hours away to come to the medical center, usually they want to go back once they hear that they no longer require care because getting family to be able to come and visit is usually a struggle.” P2

### Successes and aspirations

In addition to improved regional bed capacity management, respondents noted several other successes. Participants highlighted improvements in care delivery, increased patient and provider satisfaction, growth in available resources and services offered at affiliate sites, and improved relationships and collaboration between institutions.

I mean the biggest [success] which was shocking to me to be honest after we had done this successfully for about a year or two is we had generated, and I don't know the exact number, but a fair amount of revenue for the affiliate that was hurting a little bit. They were able to hire more nurses, open more floors, and grow programs that they weren't able to grow before. P1

The majority of participants saw opportunities for continuous improvement in collaborative care systems. Patient selection, transfer, and care delivery were described as dynamic, with aspirations for ongoing refinements.

I think something aspirational is to get a listing of which of our 12 or 13 hospitals have open beds. Who can go where, what is closer for the patient and their family as we're thinking about offloading capacity. It's not always super clear who has which beds and what kind of patients would benefit from them. I think we could also do a better job of leveraging our community partners who are not within the AMC with a dashboard and look to better divert patients. I don't think we're quite at that level yet. There have been discussions, but they haven't been instituted yet. P6

## DISCUSSION

Findings from this multicenter study offer valuable insights into how AMCs manage patient transfers to affiliated hospitals. The analysis explores the purpose, processes, barriers, facilitators, successes, and aspirations associated with these transfers. In the context of persistently high patient volumes, effectively managing system‐wide capacity is essential. Redirecting patients to affiliates with both the clinical capability and capacity ensures care is delivered safely and efficiently. A deeper understanding of these dynamics can inform strategies to optimize transfer programs, with implications for both population health management and health system operations.

Our findings are consistent with existing research on interhospital transfers, hospital consolidation, and resource allocation across health systems. Previous studies have identified similar challenges including patient selection bias, inefficiencies in transfer processes, and the impact of coordinated system‐wide efforts on patient outcomes.^6^, ^7^, ^8^, ^9^ Our interviews noted similar anecdotal concerns from the participants about patient selection bias, inefficiencies in processes, and patient outcomes; however, our study did not have discrete patient‐level data from each organization to analyze whether this was present or statistically significant. One key facilitator that emerged in our analysis was the presence of shared providers between AMCs and affiliates, which significantly enhanced communication, built trust, and supported continuity of care. Additionally, targeting specific conditions, particularly those suited for affiliate‐based specialized services or centers of excellence, highlights an opportunity to improve access to high‐quality care. In reviewing the data provided from each hospital, the successful monthly interhospital transfer volume had wide variation and did not appear to be correlated by the size of the institutions. The volume of successful transfers was more likely a reflection of the degree of efficient transfer operations and well‐coordinated collaborations between hospitals as reflected in this study rather than the size of the AMC.

Our study has several limitations. This study relies on convenience sampling and has a small sample size, which limits the generalizability of its findings. Nevertheless, the inclusion of a diverse sample of AMCs from all four major regions of the United States (Midwest, Northeast, South, and West) ranging from 550 to 1700 beds, and the application of rigorous thematic analysis lend strength to the study's validity. The findings have meaningful clinical operational implications for health systems looking to either implement or enhance their transfer programs. Tools such as standardized transfer protocols, AI‐driven decision support, and real‐time capacity tracking hold promise for improving efficiency. Equally important is the need to address equity concerns, ensuring that transfer practices do not inadvertently disadvantage specific patient populations.

## CONCLUSION

This study underscores the pivotal role of patient transfer programs in supporting high‐quality care delivery and managing capacity across healthcare systems. Successful implementation depends on addressing operational inefficiencies, promoting collaboration across institutions, and ensuring equitable access to care. A potential focus of future research could explore standardized metrics, innovative technologies like AI for optimizing transfers, and longitudinal studies to assess the long‐term impact on patient outcomes, patient experience, hospital capacity, and reimbursement.

## CONFLICT OF INTEREST STATEMENT

The authors declare no conflicts of interest.

## ETHICS STATEMENT

This study was approved by the UofM IRB HUM00225766 with exempt/not regulated status. The study adhered to the COREQ reporting guidelines for qualitative research (see attached).

## Supporting information

ATS SSI.
