# Supplementary material for: PARTNER: A qualitative study on academic and community hospitals partnerships to optimize outbound patient transfers and capacity
Source: J Hosp Med. 2025 Aug 4;21(3):239–46. doi: 10.1002/jhm.70141 (PMC12954356; doi:10.1002/jhm.70141)
Supplement: Supplementary file 1 — ATS SSI. [file JHM-21-239-s001.docx]

Appendix 1: Semi Structured interview:

On behalf of the combined University of Michigan & Dartmouth team, I would like to thank you for taking the time to answer our online survey questions regarding clinical affiliations between academic medical centers (AMCs) and community hospitals. We are excited to see that your hospitalist group has innovated in this space! **We would like to learn more about it via a recorded zoom interview, which will take about 45 minutes.** The focus of the interview will be the below questions (we are happy to extend the interview time to include sharing our experiences if helpful). *Ann Bennett, my wonderful admin is cc'd to help us schedule this appointment*. If you think someone else in your group/institution is better equipped to answer these questions, we would appreciate you replying with that information:

Thank you in advance for your contributions and we look forward to speaking to you (or a colleague) soon!

*QUESTIONS:*

1. *How many different hospital affiliations do you have that you transfer patients to?*
2. *How many patients approximately per month do you transfer to each affiliate site?*
   1. *1-10 patients per month*
   2. *10-50 patients per month*
   3. *50-100 patients per month*
   4. *100 plus*
3. *What is the available capacity/# of beds at each affiliate?*
4. *Describe the coverage model*
   1. *Are the patients cared for by the AMC hospitalists?*
   2. *Affiliate Hospitalists?*
5. *Describe the process by which patients are selected and admitted to an affiliate hospital?*
   1. *Types of clinical conditions (inclusion/exclusion criteria)*
   2. *Who triages them*
   3. *Where are they coming from (ED, home..etc)*
   4. *Patient’s home location*
   5. *Are there tools used*
   6. *Transportation*
   7. *Consent required*
   8. *Insurance coverage*
   9. *What factors impact acceptance by patients for transfer*
6. *Describe the metrics your AMC uses to capture/monitor the outcomes of the transfers? How is success defined?*
7. *Aside from outcomes discussed above, please describe additional successes/benefits achieved through these affiliate partnerships.*
   1. *Educational, research, QI*
   2. *Special population care?*
   3. *Improved institutional relationships/Worse institutional relationships*
8. *What challenges has your AMC/Affiliate operations experienced?*
   1. *Staffing*
   2. *Contract renewal*
   3. *Integration in to one or both systems*
   4. *Quality*
   5. *Recruitment*
9. *Describe any anticipated or aspirational changes in the current AMC to affiliate collaborations that exist at your institution.*
10. *Aside from managing capacity, in what other ways does your AMC collaborate with the affiliates?*
11. *Is there anything else about your AMC/community hospital affiliation that strikes you as important to tell me about?*
